# Sensitization of *Candida albicans* biofilms to various antifungal drugs by cyclosporine A

**DOI:** 10.1186/1476-0711-11-27

**Published:** 2012-10-04

**Authors:** Ravikumar B Shinde, Nitin M Chauhan, Jayant S Raut, Sankunny M Karuppayil

**Affiliations:** 1 DST-FIST Sponsored School of Life Sciences, SRTM University, Nanded, MS, 431 606, India

**Keywords:** Antifungal, Biofilms, *Candida albicans*, Calcineurine, Drug combination, Cyclosporine A, Drug resistance, Synergism

## Abstract

**Background:**

Biofilms formed by *Candida albicans* are resistant towards most of the available antifungal drugs. Therefore, infections associated with *Candida* biofilms are considered as a threat to immunocompromised patients. Combinatorial drug therapy may be a good strategy to combat *C. albicans* biofilms.

**Methods:**

Combinations of five antifungal drugs- fluconazole (FLC), voriconazole (VOR), caspofungin (CSP), amphotericin B (AmB) and nystatin (NYT) with cyclosporine A (CSA) were tested *in vitro* against planktonic and biofilm growth of *C. albicans*. Standard broth micro dilution method was used to study planktonic growth, while biofilms were studied in an *in vitro* biofilm model. A chequerboard format was used to determine fractional inhibitory concentration indices (FICI) of combination effects. Biofilm growth was analyzed using XTT-metabolic assay.

**Results:**

MICs of various antifungal drugs for planktonic growth of *C. albicans* were lowered in combination with CSA by 2 to 16 fold. Activity against biofilm development with FIC indices of 0.26, 0.28, 0.31 and 0.25 indicated synergistic interactions between FLC-CSA, VOR-CSA, CSP-CSA and AmB-CSA, respectively. Increase in efficacy of the drugs FLC, VOR and CSP against mature biofilms after addition of 62.5 μg/ml of CSA was evident with FIC indices 0.06, 0.14 and 0.37, respectively.

**Conclusions:**

The combinations with CSA re*s*ulted in increased susceptibility of biofilms to antifungal drugs. Combination of antifungal drugs with CSA would be an effective prophylactic and therapeutic strategy against biofilm associated *C. albicans* infections.

## Background

*Candida albicans* continues to be the most common fungal pathogen and a major cause of high morbidity and mortality among immunocompromised patients [1-3]. *Candida* readily forms biofilms on host tissues and medical devices implanted in the patient’s body [4]. Options of the antifungal drugs available for the treatment of systemic and invasive candidiasis are restricted to polyenes, allylamines, azoles and echinocandin class of molecules [5]. Side effects due to toxicity of the drugs and emergence of drug resistant strains have put limitations on the effective use of these drugs [5-7]. Moreover, biofilms show dramatically different properties from their planktonic counterparts, such as increased resistance to antimicrobial agents, multiple drug resistance and tolerance to host defenses. Susceptibility studies have revealed that biofilms formed by *C. albicans* may be up to 2000 times more resistant to antifungal drugs than the planktonic cells [4,8,9]. In this scenario, there is a need for new strategies to combat fungal infections, especially biofilm associated infections. Efforts are being done to explore the efficacy of combination therapy in the treatment of infections that are refractory towards antifungal drugs [10]. Combinations of antifungal drugs from different classes are being studied against *C. albicans*. Combining azoles with flucytosine was found to result in either indifference or antagonism [11,12]. Also, combinations of AmB, azoles and echinocandin with azoles/ polyenes did not show significant antifungal activity [13]. Not many studies are available which have discussed efficacy of drug combinations against biofilm growth of *C. albicans*[14]. Interestingly, results of combination against planktonic cells may not always match with that of the biofilm forms. For example, combination of FLC and AmB has synergistic effects on planktonic growth of *C. albicans* but does not alter activity of AmB against biofilms [15,16]. FLC with CSP have antagonistic effects against biofilms, unlike its planktonic counterpart [16,17]. This suggests the need for drug combination studies in biofilm settings. Various drugs including inhibitors of multidrug efflux transporters, antimicrobial agents, membrane active compounds, are being screened for their combined activity with known antifungal drugs [18]. It was found that growth of planktonic cells of *C. albicans* is sensitive to the calcineurine inhibitors FK 506 and cyclosporine A in combination with fluconazole [17]. Calcineurine is a Ca^2+^ - calmodulin activated protein phosphatase, which plays an important role in multiple aspects of fungal physiology including cation homeostasis, morphogenesis, antifungal drug resistance and virulence. Recently, it was revealed that *C. albicans* biofilms are resistant to FLC as well as CSA, however significantly sensitive to combination of the two [19]. Surprisingly, efficacy of CSA in combination with other antifungal drugs has not been studied. In our systematic study, for the first time we have analyzed the effects of CSA combination with five antifungal drugs (which belong to three classes) FLC & VOR (azoles), AmB & NYT (polyenes) and CSP (echinocandins). Efficacy of these combinations against planktonic and biofilm growth of *C. albicans* is discussed.

## Methods

### Cultures and antifungal agents

A standard strain of *Candida albicans* ATCC 90028 was obtained from the Institute of Microbial Technology, Chandigarh, India. Culture was maintained on yeast extract peptone dextrose (YPD) agar slants at 4 °C (All the media components were purchased from HiMedia laboratories Pvt. Ltd. Mumbai, India). Antifungal agents, Fluconazole (FLC) (Forcan, Cipla Ltd., India), Voriconazole (VOR) (Vonaz, United Biotech Pvt. Ltd., India), Caspofungin (CSP) (Cancidas™, Merck & Co. Inc., USA), Amphotericin B (AmB) (Lyka Pharm. Pvt. Ltd., India), Nystatin (NYT) (HiMedia, Pvt. Ltd., India), Cyclosporine A (CSA) (Sun Pharm. India Ltd., India) were obtained from local market.

### Medium and culture conditions

Activation of culture was done by inoculating a single colony from YPD agar plate (yeast extract 1 %, peptone 2 %, dextrose 2 %, agar 2 %) into 50 ml YPD broth in a 250 ml conical flask. The flasks were incubated at 30 °C at 100 rpm on an orbital shaking incubator for 24 h. Cells were harvested by centrifugation at 2000 × *g* and washed thrice with 0.1 M phosphate buffer saline (PBS), pH 7.4. Final cell number was adjusted to 1×10^7^ cells/ml and used for experiments.

### Susceptibility of planktonic cells to antifungal drugs

The susceptibility study was carried out by the standard broth micro dilution method, as per CLSI guidelines [20]. Briefly, various concentrations of drugs in the range 0.5 μg/ml to 32 μg/ml for FLC, 0.0625 μg/ml to 8 μg/ml for VOR, CSP and AmB, 0.125 μg/ml to 16 μg/ml for NYT and 7.75 to 500 μg/ml for CSA, were prepared in RPMI-1640 medium pH 7.0, (with L-glutamine, without sodium bicarbonate, buffered with 165 mM MOPS). (HiMedia laboratories Pvt. Ltd. Mumbai, India). Drugs were used in water soluble forms; hence the stock solutions were prepared freshly in RPMI medium. A series of concentrations were prepared in the 96 well plates by double dilution. Wells without drugs served as a control. Inoculum of 1×10^3^ cells/ml was added to each well and the plates were incubated at 37 °C for 48 hours and read spectrophotometrically at 620 nm using a microplate reader (Multiskan EX, Thermo Electron Corp., USA). The lowest concentration of the drugs which caused fifty percentage reduction in the absorbance compared to that of control, was considered as minimum inhibitory concentration (MIC); except for AmB where >70 % inhibition of growth was considered MIC endpoint.

### Chequerboard format for determination of FICI

Dilutions of individual drugs and their different combinations were prepared in a chequerboard format as per standard methodology [18]. A two dimensional array of serial concentrations of test compounds were used for preparation of drug dilutions. 100 μl of cell suspension was added to each well and the micro plates were incubated at 35 °C. After 48 h of incubation, absorbance was read using microplate reader at 620 nm. MIC for growth was determined as the concentrations of antifungal drugs where 50 % reduction in the absorbance compared to that of control was obtained. FIC indices were calculated with a little modification. The FICI model is expressed as: ΣFIC = FIC_A_ + FIC_B._ Where, FIC_A_ = (MIC of drug A in combination / MIC of drug A alone) FIC_B_ = (MIC of drug B in combination / MIC of drug B alone) When the value of ΣFIC ≤ 0.5, it is the synergism and when ΣFIC >4 it is known as the antagonism. A ΣFIC >0.5 but ≤ 4 is considered as indifference [21,22].

### Biofilm formation and drug susceptibility

For biofilm formation, 100 μl of the cell suspension (1×10^7^ cells/ml in PBS) was added to each well of 96 well micro plates. Plates were incubated at 37 °C on an orbital shaker for 90 minutes of adhesion phase. Wells were washed with sterile PBS to remove non-adhered cells and 200 μl of RPMI-1640 medium was added to adhered cells. To carry out susceptibility of biofilm development, medium containing various concentrations of the drugs was added at the zero hour of biofilm formation i.e. immediately after adhesion phase and the plates were incubated at 37 °C for 48 hours, at 100 rpm in an orbital shaker. While, to analyze the effects on mature biofilms, medium with a range of drug concentrations was added to the 24 hour old biofilms. The plates were further incubated for 48 hours at 37 °C [23]. Density of the cells survived in biofilm forms was analyzed through metabolic activity in XTT-formazan reduction assay.

### Biofilm quantitation by XTT assay

Biofilm formation was quantitated using XTT [i.e. 2, 3-bis (2-methoxy-4-nitro-sulfophenyl)-2H-tetrazolium-5-carboxanilide] (Sigma-Aldrich, India) reduction assay [23]. Briefly, XTT solution was prepared by mixing 1 mg/ml XTT salt in PBS and stored at −20 °C. Prior to use, menadione solution prepared in acetone (Sigma-Aldrich, India) was added to XTT to a final concentration of 4 μM. The wells containing biofilms were washed with PBS to remove non adhered cells and incubated for 5 hours in 100 μl of XTT-menadione solution in dark, at 37 °C at 100 rpm. The color formation by water soluble formazan product was measured at 450 nm using a microplate reader (Multiskan EX, Thermo Electron Corp., USA). Wells without biofilms served as a blank.

## Results

### Cyclosporine A is synergistic with antifungal drugs against planktonic forms

**Figure 1:**
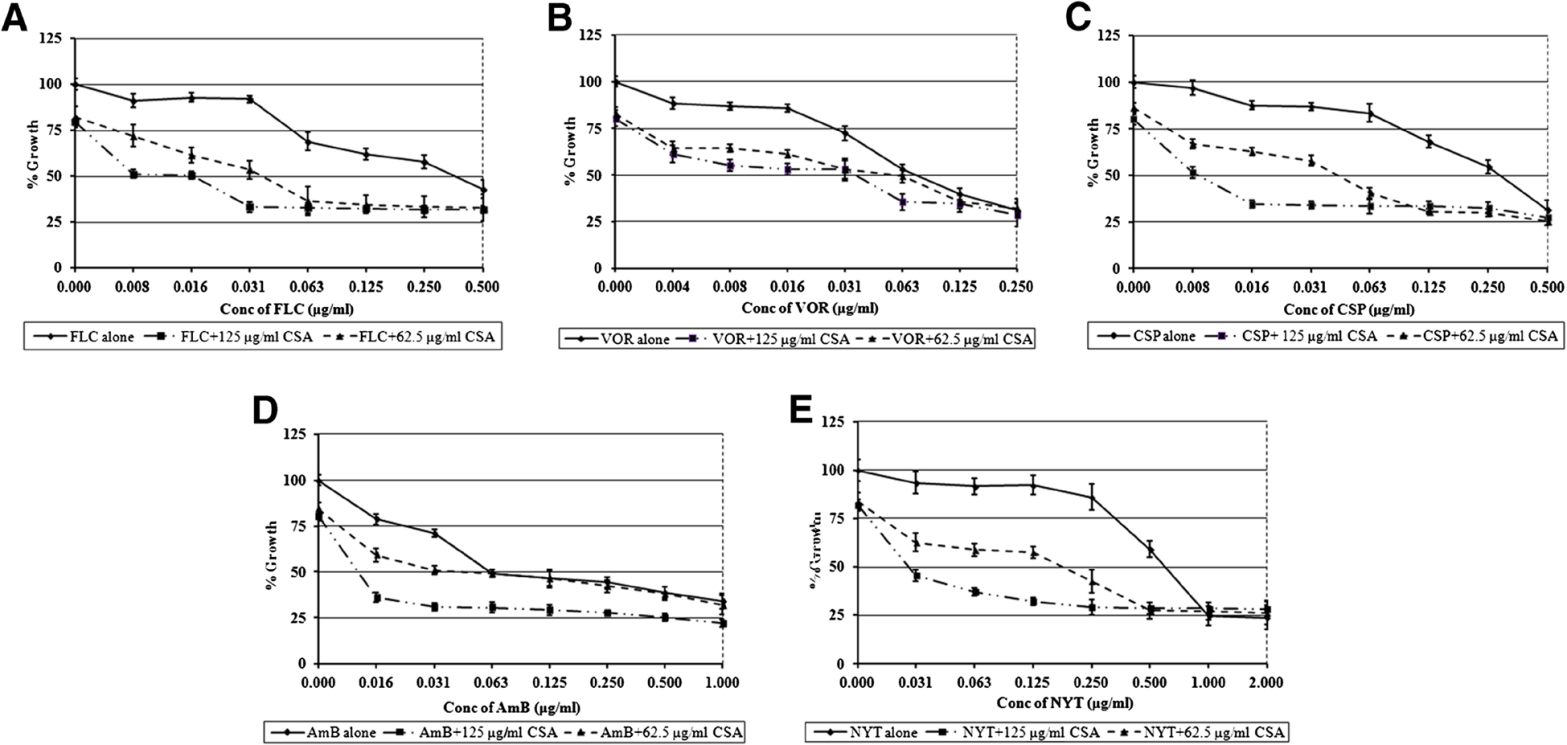
**Effect of antifungal drugs alone and in combination with Cyclosporine A, on planktonic forms of *C. albicans*, A) FLC- CSA; B) VOR- CSA; C) CSP- CSA; D) AmB- CSA; E) NYT- CSA.**

Planktonic growth of *C. albicans* was susceptible to different antifungal drugs at varying concentrations. MICs of FLC, VOR, CSP, AmB and NYT were found at 0.5 μg/ml, 0.125 μg/ml, 0.25 μg/ml, 0.5 μg/ml and 1 μg/ml, respectively (Table 1, Figure 1). CSA alone showed significant (≥50 %) inhibition of planktonic growth of *C. albicans* at concentration of 250 μg/ml (data not shown). Concentrations lower than MIC did not affect *C. albicans*, and >80 % growth was seen in presence of 62.5 and 125 μg/ml CSA (Figure 1). A significant decrease in MICs of antifungal drugs was observed when cells were treated in presence of 62.5 μg/ml of CSA. For example, MIC of FLC in combination with CSA was found to be 0.031 μg/ml i. e. sixteen times less than that of fluconazole alone. FIC index for the combination was calculated to be 0.24, which indicated that combination is synergistic. In presence of 125 μg/ml of CSA, fluconazole activity was increased by 64 times (Figure 1A). VOR concentration required to inhibit *C. albicans* in presence of 62.5 μg/ml CSA was four times less than MIC of VOR alone (Figure 1B). FIC index for this combination was calculated to be 0.37. While, 125 μg/ml of CSA increased the fluconazole activity by eight fold. Addition of 62.5 and 125 μg/ml of CSA lowered MIC of CSP to 0.063 and 0.008 μg/ml, respectively, showing 4 and 32 fold increase in susceptibility of *C. albicans* (Figure 1C). This combination was found highly synergistic with the FIC index of 0.24. Combination of 62.5 μg/ml of CSA was not effective; however AmB in presence of 125 μg/ml of CSA inhibited the growth at a concentration 32 times lesser than MIC of AmB alone (Figure 1D). In presence of 62.5 and 125 μg/ml CSA, MIC of NYT for *C. albicans* growth was obtained at 0.25 and 0.031 μg/ml, respectively. Combination of NYT and CSA was 4 to 32 times efficient than that of NYT alone (Figure 1E).

**Table 1:** **MICs of antifungals alone and in combination with CSA against *C. albicans***

**Name of Drug**	**Minimum Inhibitory Concentration (μg/ml)**					
	**Planktonic cells**^**a**^		**Biofilm development**^**b**^		**Mature biofilms**^**b**^	
	**Drug alone**	**Drug + 62.5 μg/ml CSA**	**Drug alone**	**Drug + 62.5 μg/ml CSA**	**Drug alone**	**Drug + 62.5 μg/ml CSA**
**FLC**	0.5	0.031	512	1	1024	2
**VOR**	0.125	0.031	2	0.063	4	0.061
**CSP**	0.25	0.063	0.5	0.063	1	0.125
**AmB**	0.5	0.5	0.5	0.031	0.5	0.5
**NYT**	1	0.25	3	3	3	3

^**a**^ Measured in terms of absorbance of cell density ^**b**^ Measured in terms of percentage RMA obtained in XTT assay.

### Biofilm development was sensitive to antifungal-CSA combinations

**Figure 2:**
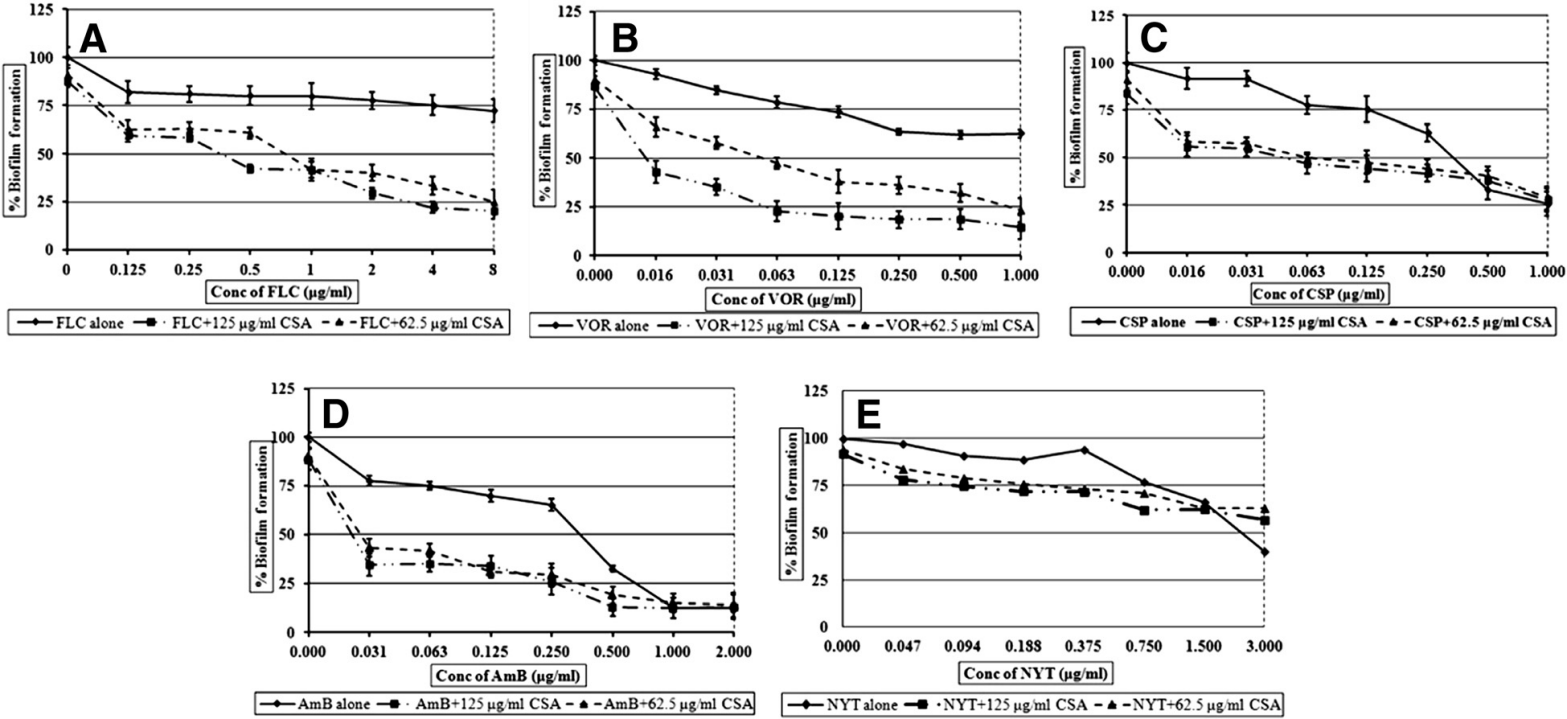
**Activity of five antifungal drugs alone and in combination with Cyclosporine A, against biofilm development of*C. albicans*.** Percentage of growth was analyzed by comparing relative metabolic activity (RMA) obtained through XTT metabolic assay. **A**) FLC- CSA; **B**) VOR- CSA; **C**) CSP- CSA; **D**) AmB- CSA; **E**) NYT- CSA.

Biofilm development was found resistant to FLC and significant inhibition was obtained only at very high concentration (i. e. 512 μg/ml). Also, VOR concentration required to inhibit biofilm formation was 16 fold high than the MIC for planktonic growth. CSA showed MIC for biofilms at 500 μg/ml concentration, whereas 62.5 and 125 μg/ml did not have any effect on biofilm development. FLC- CSA combination was found highly synergistic against biofilm development. Addition of 62.5 μg/ml of CSA sensitized the cells to fluconazole and MIC was achieved at 1 μg/ml (Table 1, Figure 2A). The FIC index for the combination was 0.26. In combination with 62.5 μg/ml of CSA, MIC of VOR was brought down to the 0.063 μg/ml (Figure 2B), with the FIC index of 0.28 indicating the synergism. While, addition of 125 μg/ml CSA showed decrease in VOR MIC for biofilm development i. e. at 0.016 μg/ml concentration. Inhibition of biofilm development was obtained at CSP concentration of 0.5 μg/ml, while combination with 62.5 and 125 μg/ml of CSA caused eight folds decrease in the MIC to make it 0.063 μg/ml (Figure 2C). A synergistic interaction between these two drugs was evident with FICI of 0.31. The combination with CSA resulted in sixteen fold decrease in concentration of the AmB required to inhibit biofilm development to give MIC at 0.031 μg/ml (Figure 2D). The FIC index of 0.25 suggested synergism between AmB and CSA. Combining NYT and CSA did not show any decrease in MIC of the individual drug (Figure 2E), and the FIC index of 0.62 indicated indifference.

### Cyclosporine A sensitizes mature biofilms to the various antifungal drugs

**Figure 3:**
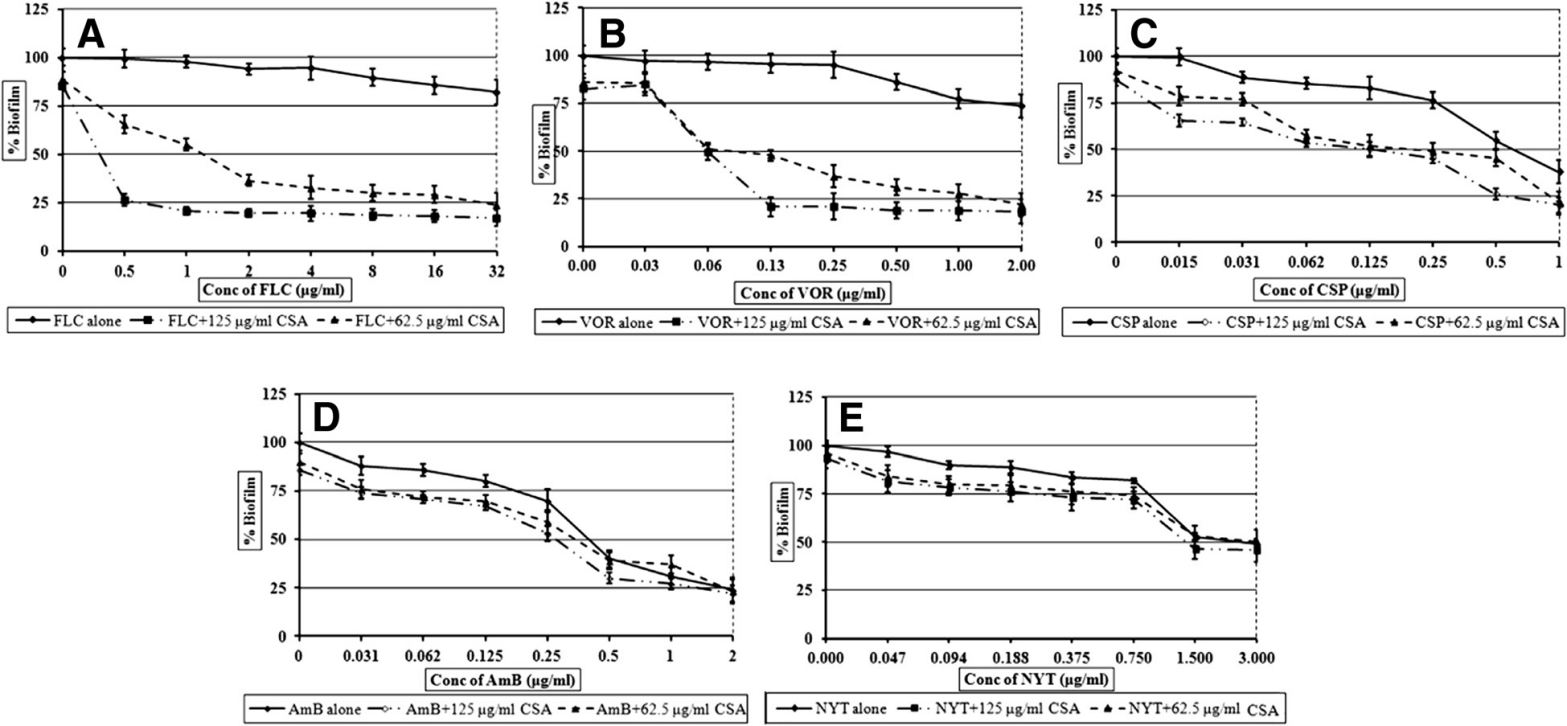
**Effect of antifungal drugs alone and in combination with Cyclosporine A, on mature biofilms of *C. albicans.*** Percentage of growth was analyzed by comparing relative metabolic activity (RMA) obtained through XTT metabolic assay. **A**) FLC- CSA; **B**) VOR- CSA; **C**) CSP- CSA; **D**) AmB- CSA; **E**) NYT- CSA.

Mature biofilms were completely resistant to FLC and VOR. MIC was obtained at very high i.e. 1024 and 4 μg/ml concentrations of FLC and VOR, respectively. FLC alone was ineffective against mature biofilms, but when combined with 62.5 μg/ml of CSA, synergistic activity (FICI of 0.06) significantly eradicated the biofilms at concentrations as low as 2 μg/ml (Table 1; Figure 3A). While, in presence of 125 μg/ml CSA, MIC of FLC was 0.5 μg/ml. Addition of CSA sensitized mature biofilms so that MIC of VOR was obtained at 0.061 μg/ml (Figure 3B). FIC index of 0.14 indicated synergism in this combination. Although CSP alone was effective in biofilm eradication, it required higher concentration compared to that of planktonic cells. One μg/ml of CSP was required to remove 50 % of biofilm. Combination of CSP-CSA caused eight fold decrease in the MIC to bring it at 0.125 μg/ml (Figure 3C). FIC index for this synergistic drug interaction was calculated to be 0.25. AmB combination with CSA showed indifference and MIC of CSA-AmB for 24 h old biofilm was found unchanged compared to AmB alone (Figure 3D). Addition of CSA did not alter the activity of NYT against biofilms. FIC indices of 0.5 and 0.62 for combinations of CSA with AmB and NYT, respectively, indicated indifference in activity.

## Discussion

Biofilm associated *C. albicans* infections among immunocompromised patients is a challenge to clinical treatment [4,8]. Various studies reported that biofilms formed by *C. albicans* are resistant to most of the commonly used antifungal drugs [10,14]. Various reasons have been proposed to be responsible for the antifungal resistance of *C. albicans* biofilms [4,8,9] including the calcinurine mediated tolerance to the drugs [17]. It is considered as a multi factorial phenomenon resulting due to surface induced gene expression. Hence, combination of drugs with different mode of action may inhibit multiple cellular targets and hence would be a good strategy against biofilms. Combinatorial approach against planktonic form of *C. albicans* has been studied and was found promising; however, only limited studies are available on biofilms [14,16,17]. Results obtained with planktonic forms may not always work in biofilm setup. Our study is of significance as CSA combination with various antifungal drugs is studied in planktonic as well as biofilm growth forms. Biofilm formation and mature biofilms were found completely resistant to the azoles, FLC and VOR, while CSP, AmB and NYT required high concentrations for preventive activity against biofilms. Resistance to FLC and VOR exhibited by early and late biofilms was totally reverted by the addition of CSA and biofilms became several folds sensitive. Combination of azoles with CSA can be developed as promising anti-biofilm strategy. Similarly increased tolerance to the drugs CSP and AmB in developing biofilms was reverted in presence of CSA, exhibiting low MIC values of CSP and AmB. Lowered antifungal concentrations may be physiologically relevant during treatment of biofilm associated infections. Addition of CSA did not alter sensitivity of mature biofilms to AmB and NYT. At 62.5 and 125 μg/ml, CSA did not have any effect on growth and viability of *C. albicans*. However, in presence of it the MICs of antifungal drugs for planktonic growth of *C. albicans* were significantly reduced, indicating ability of CSA to act as a chemosensitizer. FIC indices obtained, suggests high synergistic activity of antifungal drugs with CSA. The usefulness of FLC and CSA combinations against *C. albicans* planktonic and biofilm forms are reported by various workers [17]. However, our study for the first time demonstrates the potential of CSA against planktonic growth, biofilm formation as well as mature biofilms in combination with five antifungal drugs including FLC. Calcineurin is a Ca^2+^-calmodulin- activated phosphatase which plays important roles in various physiological functions of *C. albicans*, including morphogenesis, cell wall biosynthesis, antifungal drug resistance and virulence [17]. It is reported to play a direct role in biofilm resistance to fluconazole [17]. CSA is known as a calcineurine inhibitor. Results obtained in this study suggests that CSA mediated inhibition of biofilm specific mechanisms potentiates antifungals against drug resistant biofilms. Increased susceptibility of planktonic cells and reversal of drug resistance in biofilm growth forms after addition of CSA indicates that calcineurine may be playing important role in resistance to FLC, VOR and CSP. However, the exact mechanisms behind CSA mediated synergism with various antifungal drugs are not understood [19]. Outcome of this *in vitro* study gives insight into use of CSA- antifungal drug combinations for treatment of biofilm associated *Candida* infections. The antifungal drugs used in this study are also known for their undesirable side effects in humans, as such, reduction in their effective dosages after combination with CSA may be of wide interest. To confirm the utility of these drug combinations in clinics, *in vivo* studies and clinical trials are necessary.

## Conclusions

The results presented are the systematic information on synergistic activity of CSA with various antifungal drugs against planktonic and biofilm growth of *C. albicans*. The study for the first time showed that, combination of CSA sensitizes planktonic cells as well as drug resistant biofilms to azoles, polyene and echinocandin antifungal drugs. Further studies in this direction would give insight into an effective strategy for the prophylaxis and treatment of biofilm associated *Candida* infections.

## Abbreviations

MIC: Minimum inhibitory concentration; MFC: Minimum fungicidal concentration; ATCC: American type culture collection; MOPS: Morpholinepropanesulfonic acid; FLC: Fluconazole; VOR: Voriconazole; CSP: Caspofungin; AmB: Amphotericin B; NYT: Nystatin; CSA: Cyclosporine A; CLSI: Clinical Laboratory Standards Institute; FICI: Fractional inhibitory concentration index; XTT: 2, 3-bis (2-methoxy-4-nitro-5-sulfo-phenyl)-2H-tetrazolium-5-carboxanilide.

## Competing interests

The authors declare that they have no competing interests.

## Authors' contributions

RBS carried out all experimental work, data acquisition and analysis, literature search. NMC and JSR contributed to experiments, writing and manuscript preparation. SMK was responsible for study concept, designing and coordinating the research, supervising the work and revising the manuscript. All authors read and approved the final manuscript.
